# Comparison of serum levels of vitamin D in periodontitis patients with and without type 2 diabetes and healthy subjects

**DOI:** 10.1002/cre2.657

**Published:** 2022-10-31

**Authors:** Narjes Akbari, Marjan Hanafi Bojd, Mahdjoube Goldani Moghadam, Vajehallah Raeesi

**Affiliations:** ^1^ School of Dentistry Birjand University of Medical Sciences Birjand Iran; ^2^ School of Medicine Birjand University of Medical Sciences Birjand Iran

**Keywords:** periodontitis, type 2 diabetes, vitamin D

## Abstract

**Objective:**

The purpose of this study is to find out the levels of serum vitamin D in periodontitis patients with and without type 2 diabetes and to compare them with healthy subjects.

**Methods:**

In this study, 48 periodontitis patients with type 2 diabetes mellitus (PDM), 53 periodontitis patients (P), and 42 controls who were in the age ranges of 30–50 years and had the entry requirements were selected. Plaque index, calculus index, modified gingival index, pocket depth, clinical attachment loss (CAL), and tooth loss were measured. Serum 25(OH)D level was estimated by electrochemiluminescence immunoassay, and fasting blood sugar and glycosylated hemoglobin were estimated by biochemical colorimetric assays.

**Results:**

The mean serum 25(OH)D level was 17.06 ± 10.73, 15.12 ± 7.74, and 14.17 ± 11.04 ηg/ml for PDM, P, and control groups, respectively, showing no statistical difference. The mean CAL was significantly high in diabetic patients as compared to other groups. Prediabetes prevalence was significantly high in periodontitis patients as compared to controls.

**Conclusions:**

The prevalence of vitamin D insufficiency was high in the population studied. Serum levels of 25(OH)D showed no significant difference between groups. Periodontitis patients showed an elevated risk for diabetes.

## 1 INTRODUCTION

Periodontitis is a chronic multifactorial inflammatory disease caused by the host's immune response to the accumulation of bacterial plaque and biofilm leading to periodontal destruction (Newman et al., 2015; Wu et al., 2020). Inflammation of the gingival tissues is the first result of the accumulation of bacterial biofilm on the tooth surfaces. It could then be developed into periodontitis if remained untreated or in susceptible individuals (Newman et al., 2015). Factors like diabetes or vitamin D deficiency are known to intensify inflammatory responses, which leads to more periodontal destruction (Genco & Borgnakke, 2000).

Type 2 diabetes is among the most common risk factors of periodontitis so periodontitis is considered the sixth complication of diabetes. Diabetes by creating changes in host defense, collagen metabolism, as well as vascular changes, leads to a more inflammatory response to pathogens of periodontitis (Cerda et al., 1994; Leite et al., 2013).

Vitamin D as a vital nutrient in bone strength and prevention of osteoporosis plays an important role in chronic inflammatory diseases and has a potential anti‐inflammatory effect. Vitamin D deficiency can create a favorable condition for the development of chronic diseases, such as diabetes, high blood pressure, cancers, and chronic inflammatory diseases like periodontitis (Hiremath et al., 2013). Oral epithelial cells are capable of converting vitamin D into its active form, which can lead to the expression of host defense mediators and thus increased defense against pathogenic bacteria of periodontal disease (Abreu et al., 2016). In addition, vitamin D plays an important role in regulating insulin activity and direct stimulation of the genes of the insulin receptor. Several cross‐sectional studies have shown a reverse relationship between serum 25(OH)D concentrations and insulin resistance (Grammatiki et al., 2017).

Understanding controllable risk factors for chronic periodontitis in diabetic patients is useful for the prevention and also treatment of periodontitis, which may lead to lower tooth loss and development of oral health. Few studies have been conducted to determine the serum levels of this vitamin in the presence of both diabetes and periodontitis. This study aimed to compare the levels of serum vitamin D in periodontitis patients with and without type 2 diabetes with healthy subjects.

## 2 MATERIALS AND METHODS

This case–control study was conducted at Valiasr hospital of Birjand, Iran. The study was approved by the Birjand University of Medical Sciences ethics committee.

Subjects between the ages of 30 and 50 years among those referring to the clinics of the hospital who had the entry requirements, and after their informed written consent, entered the study. After evaluating a total number of 165 subjects, 143 subjects were selected including 48 periodontitis patients with type 2 diabetes mellitus (19 males, 29 female), 53 periodontitis patients (22 males, 31 female), and 42 controls (9 males, 33 female).

The diagnostic criteria for chronic periodontitis are based on the presence and extent of gingival inflammation, frequently measured as bleeding no probing, pocket depth (PD), clinical attachment loss (CAL), and radiographically assessment of the pattern and extent of alveolar bone loss. Radiographs have not been the predominant measurement in epidemiologic studies because of radiation exposure and technical problems (Page & Eke, 2007). Subjects with significant CAL and periodontal pocket along with gingival inflammation observed in the clinical examination were considered periodontitis patients, and those who had a healthy periodontium according to the diagnostic criteria were considered nonperiodontitis subjects.

Type 2 diabetes diagnostic criteria were based on its valid criterion of serum blood glucose of at least 200 mg/dl, fasting blood sugar (FBS) of at least 126 mg/dl, 2 h glucose levels of at least 200 mg/dl, or glycosylated hemoglobin (HbA1c) of at least 6.5%.

Subjects with any history of smoking, antibiotic treatment within the last 3 weeks, periodontal treatment within the last 6 months, presence of renal or cardiovascular diseases, malignancies, multiple sclerosis, or bone diseases that are associated with vitamin D deficiency, postmenopausal women, and pregnant women were excluded from the study.

Knowing that periodontitis and type 2 diabetes do not seem to occur in only specific age ranges, according to the slow progression of the periodontitis, its clinical signs may not appear before the age of 30. Similarly, type 2 diabetes, is usually seen in older adults (2008). On the other hand, hormonal changes in women older than 50 years, can increase the risk of osteoporosis and vitamin D deficiency (Tandon et al., 2014). Therefore, to eliminate the effect of age on serum levels of vitamin D, study subjects were selected from the age range of 30–50 years old.

The sample size was calculated based on the study performed by Rosamma Joseph et al. who evaluated levels of serum vitamin D in chronic periodontitis patients with type 2 diabetes mellitus, based on the comparison of the mean vitamin D in the chronic periodontitis group, with the control group, 17 subjects in each group were calculated. We estimated a larger sample size of 50 subjects for each group (Kelishadi et al., 2016).

A questionnaire including gender, age, educational level, job, family history of diabetes, history of medication and systemic diseases, smoking habits, duration of daily sun exposure, and history of any vitamin D supplement intake was completed by an interviewer. Then oral hygiene and periodontal status were assessed by measuring Silness and Löe simplified plaque index (PI), Greene and Vermillion simplified calculus index (CI), Lobene et al. modified gingival index (MGI), PD, CAL, and tooth loss. CAL was measured from the cementoenamel junction to the base of the clinical gingival pocket, and PD was defined as the distance from the gingival margin to the base of the clinical gingival pocket. Periodontal indices were measured for all teeth, except the third molar, on six sites (mesio‐buccal, mid‐buccal, disto‐buccal, mesio‐lingual, mid‐lingual, and disto‐lingual) using William's graduated periodontal probe by a single trained dental student.

The severity of periodontitis can be described as mild, moderate, or severe. Mild periodontitis is defined as having CAL of 1–2 mm, moderate periodontitis describes CAL of 3–4 mm, and severe form is defined as CAL of 5 mm or more (Newman et al., 2015). Based on the number of teeth with CAL, the distribution of periodontitis can be classified into two following categories: localized periodontitis with less than 30% of the teeth showing attachment loss and generalized one meaning that 30% or more of the teeth show CAL (Newman et al., 2015).

The subjects then were referred to the laboratory to assess FBS, glycosylated hemoglobin (HbA1c), and 25(OH)D serum levels. A total of  2.5 ml of blood was collected per each subject in the morning after a 10–12 h overnight fast, containing 1 ml of blood was collected into ethylenediamine tetraacetic acid anticoagulant containing tubes for glycosylated hemoglobin (HbA1c) and 1.5 ml for FBS and 25(OH)D. Then the blood was centrifuged at 4000 rpm for 10 min at room temperature. FBS and HbA1c were determined by biochemical colorimetric method using a diagnostic kit of BIONIK company, and 25(OH)D was assessed by electrochemiluminescence immunoassay in a Roche COBAS E411 analyzer.

Vitamin D deficiency is defined as a serum 25(OH)D level below 20 ng/ml and vitamin D insufficiency as less than 30 ng/ml (75 nmol/L) (Gallagher & Sai, 2010). To have beneficial effects on health, serum levels of vitamin D should reach 30 ng/ml (Holick & Chen, 2008).

## 3 STATISTICAL ANALYSIS

A comparison of mean vitamin D levels based on vitamin D supplementation was tested by Kruskal–Wallis test. For comparison of periodontal parameters, analysis of variance (ANOVA) and Kruskal–Wallis test were used. Fisher's exact test and *χ* ^2^ test were used to the comparison of vitamin D status in study groups. Comparison of levels of vitamin D based on the severity of periodontitis was tested by ANOVA test. Data were analyzed by SPSS‐22 software. Significance was considered less than .05.

## 4 RESULTS

In this study, 48 periodontitis patients with type 2 diabetes mellitus (19 males, 29 female), 53 periodontitis patients (22 males, 31 female), and 42 controls (9 males, 33 female) with mean ages of 43 ± 4.64, 39 ± 6.49, and 36 ± 5 years, respectively, were evaluated according to taking vitamin D supplements and in two different age groups (30–40 and 40–50). The distribution of percentages of educational level and age group were different in these groups, but the percentage distribution of gender was similar among them (Table 1). In those without taking any vitamin D supplements, the mean levels of 25(OH)D were 27.7 ± 11.7, 24.2 ± 6.8, and 19.2 ± 3.2 ng/ml for periodontitis patients with type 2 diabetes mellitus, periodontitis patients, and controls, respectively (Table 2). To remove the confounding effect of age, the study population was stratified into two age categories (30–40/40–50 years). In both age categories, the mean serum vitamin D levels were not significantly different among our three study groups. Mean PI, MGI, and PD showed higher scores among periodontitis patients in comparison to the other groups, whereas mean CAL and CI had higher scores for periodontitis patients with type 2 diabetes mellitus (Table 3). According to Table 3, mean tooth loss was found to be 5.2 ± 5.04, 4.05 ± 4.2, and 1.5 ± 2.2 for periodontitis patients with type 2 diabetes mellitus, periodontitis patients, and controls, respectively (*p* < .001).

**Table 1:** Demographic data

Parameters	PDM ($n_{1}$= 48)	*p* ($n_{2}$= 53)	Controls ($n_{3}$= 42)	*p*
Age (% within the group)				<.001
30–40	25	54.7	83.3	
40–50	75	45.3	16.7	
Distribution of education status (% within the group)				.01
Illiterate	10.4	1.9	2.4	
Below diploma	54.2	67.9	40.5	
Diploma	10.4	9.4	31	
Higher educational level than a diploma	25	20.8	26.2	
Gender (% within the group)				.08
Male	39.6	41.5	21.4	
Female	60.4	58.5	78.6	

*Note*: *p* ˂ .05 is considered statistically significant.

Abbreviations: P, periodontitis patients; PDM, periodontitis patients with type 2 diabetes mellitus.

**Table 2:** Comparison of mean serum vitamin D levels in subjects studied based on vitamin D supplementation.

Vitamin D supplementation	Group	Serum levels of vitamin D (mean ± SD)	*p*
Subjects taking vitamin D supplements	PDM ($n_{1}$= 24)	17.06 ± 10.7	*p* = .38
	P ($n_{2}$= 7)	15.1 ± 7.7	
	controls ($n_{3}$= 9)	14.1 ± 11.04	
Subjects not taking vitamin D supplements	PDM ($n_{1}$= 24)	27.7 ± 11.7	*p* = .09
	P ($n_{2}$= 46)	24.2 ± 6.8	
	controls ($n_{3}$= 33)	19.2 ± 3.2	

*Note*: *p* ˂ .05 is considered statistically significant.

Abbreviations: P, periodontitis patients; PDM, periodontitis patients with type 2 diabetes mellitus; SD, standard deviation.

**Table 3:** Comparison of periodontal parameters between study groups

Periodontal index	Group	Mean ± SD	Minimum	Maximum	Statistical test
Plaque index	PDM ($n_{1}$= 48)	1.29 ± 0.52	0.10	2.35	*p* ˂ .001
	Periodontitis patients ($n_{2}$= 53)	1.59 ± 0.41	0.59	2.49	
	Controls ($n_{3}$= 42)	1.19 ± 0.39	0.25	1.84	
Calculus index	PDM ($n_{1}$= 48)	0.22 ± 0.17	0	0.70	*p* = .43
	Periodontitis patients ($n_{2}$= 53)	0.21 ± 0.22	0	1.46	
	Controls ($n_{3}$= 42)	0.08 ± 0.07	0	1.46	
Modified gingival index	PDM ($n_{1}$= 48)	0.51 ± 0.39	0	1.57	*p* = .06
	Periodontitis patients ($n_{2}$= 53)	0.71 ± 0.57	0.11	2.51	
	Controls ($n_{3}$= 42)	0.39 ± 0.39	0	2.09	
Pocket depth	PDM ($n_{1}$= 48)	2.06 ± 0.29	1.32	2.58	*p* ˂ .001
	Periodontitis patients ($n_{2}$= 53)	2.27 ± 0.24	1.68	2.85	
	Controls ($n_{3}$= 42)	2.00 ± 0.30	1.22	2.55	
Clinical attachment loss	PDM ($n_{1}$= 48)	0.56 ± 0.49	0.04	2.16	*p* = .06
	Periodontitis patients ($n_{2}$ = 53)	0.32 ± 0.31	0.05	1.46	
	Controls ($n_{3}$= 42)	0 ± 0	0	0	
Tooth loss	PDM ($n_{1}$= 48)	5.29 ± 5.04	0	20	*p* ˂ .001
	Periodontitis patients ($n_{2}$= 53)	4.05 ± 4.28	0	21	
	Controls ($n_{3}$ = 42)	1.52 ± 2.29	0	10	

*Note*: *p* ˂ .05 is considered statistically significant.

Abbreviations: SD, standard deviation; PDM, periodontitis patients with type 2 diabetes mellitus.

To eliminate the effect of supplementation, we used classification analysis, which showed no significant relationship between vitamin D and periodontitis and diabetes in those taking the supplement and those without supplementation (Table 4).

**Table 4:** Comparison of vitamin D status in study groups based on taking vitamin D supplementation

Taking vitamin D supplementation	Group	Vitamin D status			*p*
		Normal	*i*nsufficient	Deficient	
Yes	PDM ($n_{1}$ = 24)	8 (33.3%)	16 (66.7%)	0 (0%)	*p* = .14
	Periodontitis patients ($n_{2}$ = 7)	2 (28.6%)	5 (71.4%)	0 (0%)	
	controls ($n_{3}$= 9)	0 (0%)	9 (100%)	0 (0%)	
No	PDM ($n_{1}$= 24)	2 (8.3%)	15 (62.5%)	7 (29.2%)	*p* = .14
	Periodontitis patients ($n_{2}$= 46)	1 (2.2%)	32 (69.6%)	13 (28.3%)	
	controls ($n_{3}$= 33)	3 (9.1%)	14 (42.4%)	16 (48.5%)	
Total	PDM ($n_{1}$= 48)	10 (20.8%)	31 (64.6%)	7 (14.6%)	*p* = .01
	Periodontitis patients ($n_{2}$ = 53)	3 (5.7%)	37 (69.8%)	13 (24.5%)	
	controls ($n_{3}$ = 42)	3 (7.1%)	23 (54.8%)	16 (38.1%)	

According to blood tests, FBS levels in the diabetic group ranged from at least 87 mg/dl to a maximum of 391 mg/dl and had an average of 188.68 ± 68.03 mg/dl. Glycated hemoglobin (HbA1C) levels in the diabetic group showed a minimum of 5.2 and a maximum of 13.8 with an average of 8.3 ± 1.91. The duration of diabetes since diagnosis ranged from 2 months to 20 years and was on average 5.73 ± 7.57 years.

The number of nondiabetic periodontitis patients with HbA1c levels of 5.7%–6.4% was 13 (24.5%), whereas only 3 (7.1%) of controls showed these levels. The prevalence of prediabetes in periodontitis patients was significantly higher than in the control group (Table 5).

**Table 5:** Comparison of HbA1C levels between periodontitis patients and control group

	HbA1C			
	≤5.6%	5.7%–6.4%	≥6.5%	*p*
Periodontitis patients ($n_{1}$ = 53)	40 (75.5%)	13 (24.5%)	0 (0%)	*p* = .04
Controls ($n_{2}$= 42)	38 (90.5%)	3 (7.1%)	1 (2.4%)	
Total	78 (82.1%)	16 (16.8%)	1 (1.1%)	

A comparison of serum levels of vitamin D based on the severity of periodontitis showed that the mean serum level of vitamin D was not significantly different in terms of periodontitis severity in diabetic and nondiabetic periodontitis patients (Table 6).

**Table 6:** Comparison of serum levels of vitamin D based on the severity of periodontitis

	Severity of periodontitis	Vitamin D level mean ± SD	Minimum level of vitamin D (ng/ml)	Maximum level of vitamin D (ng/ml)	ANOVA test
Periodontitis patients with type 2 diabetes mellitus	Mild ($n_{1}$= 13)	20.24 ± 8.86	3.37	33.10	*p* = .76
	Moderate ($n_{2}$= 25)	23.39 ± 13.20	4.24	64.63	
	Severe ($n_{3}$= 10)	22.77 ± 14.77	2.67	46.19	
Periodontitis nondiabetic patients	Mild ($n_{1}$ = 28)	14.82 ± 7.98	2.80	34.20	*p* = .17
	Moderate ($n_{2}$= 21)	18.90 ± 8.23	7.80	40.83	
	Severe ($n_{3}$= 4)	13.47 ± 7.65	5.96	20.30	

Abbreviation: ANOVA, analysis of varience.

## 5 DISCUSSION

The present study did not report a strong association between vitamin D status and severity of periodontitis in diabetic and nondiabetic patients. Also, serum levels of vitamin D did not show any significant difference in chronic periodontitis patients with type 2 diabetes mellitus in comparison to chronic periodontitis patients and healthy subjects.

Several studies have been conducted evaluating the association between serum levels of vitamin D and diabetes and also periodontitis (Machado et al., 2020; Perić et al., 2018), but there are few studies exploring vitamin D levels in type 2 diabetic patients with chronic periodontitis (Joseph et al., 2015). The mean serum vitamin D level of the studied population was insufficient, which is in line with epidemiologic studies on the high prevalence of vitamin D deficiency in Iran (Ebrahimi et al., 2014; Kelishadi et al., 2016; Tabrizi et al., 2017). The high prevalence of vitamin D deficiency in Iran could be due to inadequate exposure to sunlight and an inappropriate diet.

Evaluation of serum levels of vitamin D in subjects without receiving any vitamin D supplement showed no significant difference between the three study groups. It is following some previous studies (Al‐Shoumer et al., 2013; Kafeshani et al., 2016). A similar study showed lower levels of 25(OH)D in type 2 diabetic patients with chronic periodontitis in comparison to nondiabetic chronic periodontitis patients, and a greater level in healthy subjects (Joseph et al., 2015). Similarly, Taherani et al. showed lower levels of 25(OH)D in type 2 diabetic patients in comparison to their nondiabetic counterparts (Tahrani et al., 2010). These discrepancies may be due to the lower sample size of our study. Another reason may be the differences in geographic areas where the studies were conducted since our study, similar to Kafeshani's study, was conducted in Iran, where a high prevalence of vitamin D deficiency is present in all study groups (Kafeshani et al., 2016). The high prevalence of vitamin D deficiency in the studied population weakens the possibility of a significant difference in serum levels of vitamin D between study groups. Another factor influencing vitamin D status is obesity and body mass index (BMI), which is not considered in our study. Obesity can be accompanied by an inappropriate diet and insufficient intake of sunlight due to a lack of mobility. If the control group has a higher BMI, it can show a lower serum level of vitamin D in comparison with its counterparts (Baradaran et al., 2012; Wortsman et al., 2000). The discrepancy between the present study and its similar study conducted by Joseph et al. maybe because the mild type of chronic periodontitis, like its moderate and severe form, were counted in our periodontitis patients, while in the study of Joseph et al. mild form of periodontitis was considered as healthy periodontium (Joseph et al., 2015).

Age, skin melanin content, time and duration of daily sunlight exposure, season, and latitude are also known to affect vitamin D status. The consumption of fish, as well as daily sun exposure, can provide the daily requirement of vitamin D for a normal adult (Vieth, 2011).

The comparison of periodontal indices showed a higher mean of CAL in diabetics, while PD and PI had higher scores among the nondiabetic chronic periodontitis group. A meta‐analysis study showed greater severity of periodontal diseases among diabetics in comparison to nondiabetic subjects (Khader et al., 2006). Diabetes is associated with an increased risk of periodontitis in adults (Nascimento et al., 2018). There are several mechanisms to explain this relationship, including changes in host defense, collagen metabolism, and vascular changes in diabetics. Subjects with uncontrolled type 2 diabetes mellitus, show stronger inflammatory responses to periodontal bacteria. Since insulin can reduce the levels of inflammatory interleukin‐6 mediators in the acute inflammatory response, insulin resistance acts as an effective factor in increasing the concentration of inflammatory mediators, which leads to more periodontal tissue destruction (Chee et al., 2013; Leite et al., 2013; Wu et al., 2020).

Prediabetes, which is known as HbA1C levels of 5.7%–6.4%, is associated with a higher risk of diabetes. The present study showed a significantly higher prevalence of prediabetes among nondiabetic chronic periodontitis patients in comparison to controls, in line with the study of Demmer et al., which reported a two‐fold higher risk of diabetes among moderate periodontitis patients than their healthy counterparts (Demmer et al., 2008).

## 6 CONCLUSION

Although serum vitamin D levels were different in the studied groups, after eliminating the effect of taking vitamin D supplementation, it was found that the mean serum vitamin D level was not different between the groups.

The prevalence of prediabetes in patients with periodontitis is higher than in healthy people.

It is suggested that this study be conducted with larger sample size and in addition to matching the age and sex of individuals, severity of periodontitis, and other conditions affecting blood vitamin D levels, including BMI, place of living, skin pigmentation, and so on, should also be taken into consideration.

## AUTHOR CONTRIBUTIONS

Narjes Akbari, Marjan Hanafi Bojd, and Vajehallah Raeesi designed and conceived the study. Narjes Akbari and Marjan Hanafi Bojd collected the data. Narjes Akbari, Marjan Hanafi Bojd, and Mahdjoube Goldani Moghadam analyzed and interpreted the data. Narjes Akbari, Marjan Hanafi Bojd, and Vajehallah Raeesi drafted the manuscript. Vajehallah Raeesi and Mahdjoube Goldani Moghadam provided administrative, technical, and material support. All authors contributed to the article and approved the submitted version.

## CONFLICT OF INTEREST

The authors declare that the research was conducted in the absence of any commercial or financial relationships that could be construed as a potential conflict of interest.

## Data Availability

The data presented in this study are available on request from the corresponding author.
